# (2,9-Dimethyl-1,10-phenanthroline-κ^2^ *N*,*N*′)bis­(thio­cyanato-κ*S*)mercury(II)

**DOI:** 10.1107/S1600536812038160

**Published:** 2012-09-15

**Authors:** Ismail Warad, Taibi Ben Hadda, Belkheir Hammouti, Salim F. Haddad

**Affiliations:** a Department of Chemistry, College of Science, King Saud University, PO Box 2455 Riyadh 11451, Saudi Arabia; b Laboratoire LCM, Faculté Sciences, Université Mohammed Ier, Oujda 60000, Morocco; c LCAE–URAC18, Faculté des Sciences, Université Mohammed Ier, Oujda 60000, Morocco; d Department of Chemistry, The University of Jordan, Amman 11942, Jordan

## Abstract

The asymmetric unit of the title compound, [Hg(SCN)_2_(C_14_H_12_N_2_)], contains two complex mol­ecules in which the Hg^II^ atoms are both four-coordinated in a distorted tetra­hedral configuration by two N atoms from a chelating 2,9-dimethyl-1,10-phenanthroline ligand and by two S atoms from two thio­cyanate anions. The 1,10-phenanthroline ligand is slightly folded for one complex, the dihedral angle between the pyridine planes being 5.3 (1)°. In contrast it is nearly planar [0.5 (1)°] as it complexes with the other Hg^II^ atom. The thio­cyanate ligands are virtually linear and the S atom is bonded to Hg^II^ with N⋯S—Hg angles ranging from 99.3 (1) to 103.5 (1)°. Despite the presence of six aromatic rings in the asymmetric unit, there are no significant inter­molecular π–π contacts between phenanthroline ligands as the centroid–centroid distance of the closest contact between six-membered rings is 4.11 (1) A°.

## Related literature

For the coordination geometry of other complexes with C_14_H_12_N_2_, see: Alizadeh *et al.* (2009 ▶); Wang & Zhong (2009 ▶); Warad *et al.* (2011 ▶). For therapeutic applications of similar compounds, see: Miller *et al.* (1999 ▶); Lange *et al.* (2000 ▶); Bodoki *et al.* (2009 ▶).
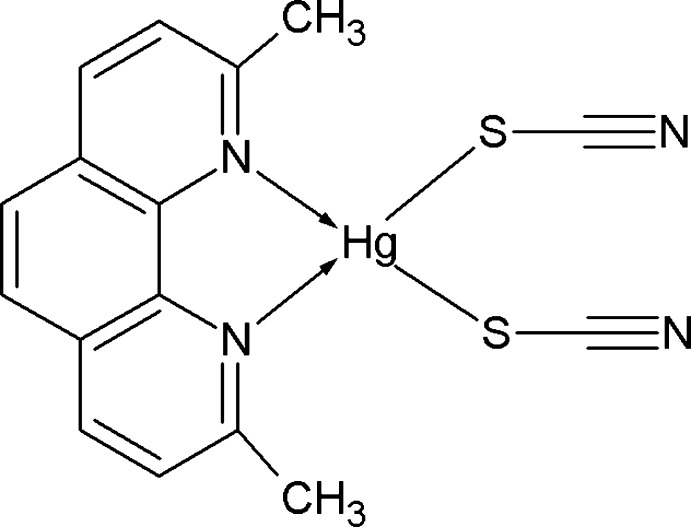


## Experimental

### 

#### Crystal data

[Hg(NCS)_2_(C_14_H_12_N_2_)]
*M* *_r_* = 525.01
Triclinic, 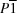

*a* = 8.1593 (4) Å
*b* = 11.2985 (5) Å
*c* = 18.9456 (9) Å
α = 77.205 (4)°
β = 84.015 (4)°
γ = 89.802 (4)°
*V* = 1693.55 (14) Å^3^
*Z* = 4
Mo *K*α radiation
μ = 9.34 mm^−1^
*T* = 293 K
0.40 × 0.20 × 0.15 mm

#### Data collection

Agilent Xcalibur Eos diffractometer
Absorption correction: multi-scan (*CrysAlis PRO*; Agilent, 2010 ▶) *T*_min_ = 0.122, *T*_max_ = 0.246
11206 measured reflections
5985 independent reflections
4876 reflections with *I* > 2σ(*I*)
*R*_int_ = 0.041

#### Refinement

*R*[*F* ^2^ > 2σ(*F* ^2^)] = 0.033
*wR*(*F* ^2^) = 0.066
*S* = 1.02
5985 reflections
419 parameters
H-atom parameters constrained
Δρ_max_ = 0.65 e Å^−3^
Δρ_min_ = −1.13 e Å^−3^

### 

Data collection: *CrysAlis PRO* (Agilent, 2010 ▶); cell refinement: *CrysAlis PRO*; data reduction: *CrysAlis PRO*; program(s) used to solve structure: *SHELXS97* (Sheldrick, 2008 ▶); program(s) used to refine structure: *SHELXL97* (Sheldrick, 2008 ▶); molecular graphics: *ORTEPIII* (Burnett & Johnson, 1996 ▶); software used to prepare material for publication: *SHELXL97*.

## Supplementary Material

Crystal structure: contains datablock(s) I, global. DOI: 10.1107/S1600536812038160/vn2049sup1.cif

Structure factors: contains datablock(s) I. DOI: 10.1107/S1600536812038160/vn2049Isup2.hkl

Additional supplementary materials:  crystallographic information; 3D view; checkCIF report

## Figures and Tables

**Table 1:** Selected bond lengths (Å)

Hg1—N1	2.396 (4)
Hg1—N2	2.395 (4)
Hg1—S1	2.4201 (16)
Hg1—S2	2.4488 (16)
Hg2—N5	2.384 (4)
Hg2—N6	2.362 (4)
Hg2—S3	2.4741 (16)
Hg2—S4	2.4013 (18)

**Fig. 1.**
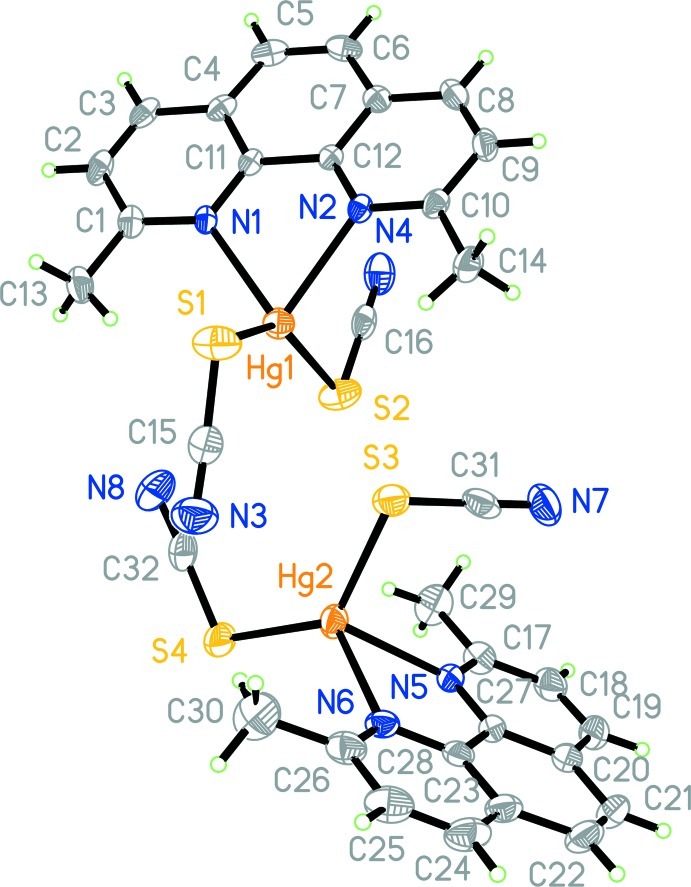
The title compound with displacement ellipsoids drawn at the 30% probability level. H atoms are shown as spheres of arbitary radius.

## supplementary crystallographic information

### Comment

Transition metal complexes using 1,10-phenanthroline (*phen*) and their modified derivatives as ligands are particularly attractive species for the design and development of novel diagnostic and therapeutic agents, that can recognize and selectively cleave DNA (Miller *et al.*, 1999; Bodoki *et al.*, 2009). The reaction of Hg(SCN)_2_, with *dmphen* = 2,9-dimethyl-1,10-phenanthroline ligand yields Hg(SCN)_2_(*dmphen*) mixed ligand complexes. The number of ligands bound to the metal cation is influenced greatly by both the chemistry and geometry of ligand and the type of co-ligand SCN (Lange *et al.*, 2000). Here we report the synthesis and crystal structure of a new Hg^II^ complex, [Hg(SCN)_2_(dmphen)].

The molecular structure of Hg(SCN)_2_(*dmphen*), along with the numbering scheme, is shown in Fig. 1. The two Hg^II^ cations are located on general positions and coordinated to two nitrogen atoms of one *dmphen* bidentate ligand and two SCN ions. A similar coordination geometry around the central atom has been observed in other metal complexes involving the same *dmphen* ligand such as [HgBr_2_(dmphen)] (Alizadeh *et al.*, 2009), [CuCl_2_(dmphen)] (Wang & Zhong, 2009), [CdI_2_(dmphen)] (Warad *et al.*, 2011), and [CdBr_2_(dmphen)] (Warad *et al.*, 2011).

One of the two 2,9-dimethyl-1,10-phenanthroline ligands, the one bonded to Hg1, is folded by 5.3 (1)° while the other bonded to Hg2 is planar. Such conjugate double bond systems are expected to be planar. The probable reason comes from packing considerations. The soft Hg bonds to the soft S atom of SCN^-^ as expected. the variations in the approach angle, 99.3 (1) to 103.5 (1)° should also be attributed to packing considerations.

### Experimental

The title compound was prepared by a procedure similar to that used for [CdI_2_(dmphen)] (Warad *et al.*, 2011). A mixture of mercury thiosyanode (Hg(SCN)_2_, 50 mg, 0.16 mmol) in methanol (10 ml) and *dmphen* (32.8 mg, 0.16 mmol) in dichloromethane (5 ml) is stirred for 2 h at room temperature. The obtained solution was concentrated to about 1 ml under reduced pressure and mixed to 40 ml of *n*-hexane. This caused the precipitation of a white powder of 75 mg, (90% yield) which was filtered, dried and used for the preparation of colorless prisms of [Hg(SCN)_2_(*dmphen*)] by slow diffusion of *n*-hexane into a solution of the complex in dichloromethane. All chemicals were purchased from Acros/Belgium.

### Refinement

All nonhydrogen atoms were refined anisotropically. H atoms were positioned geometrically, with C—H = 0.93 and 0.96 Å for aromatic and methyl H, respectively, and constrained to ride on their parent atoms, with *U*_iso_(H) = 1.2*U*_eq_(C) except for methyl groups where *U*_iso_(H)= 1.5*U*_eq_(C).

### Crystal data

**Table:**

[Hg(NCS)_2_(C_14_H_12_N_2_)]	*Z* = 4
*M**_r_* = 525.01	*F*(000) = 992
Triclinic, *P*1	*D*_x_ = 2.059 Mg m^−^^3^
Hall symbol: -P 1	Mo *K*α radiation, λ = 0.71073 Å
*a* = 8.1593 (4) Å	Cell parameters from 5117 reflections
*b* = 11.2985 (5) Å	θ = 3.1–29.2°
*c* = 18.9456 (9) Å	µ = 9.34 mm^−^^1^
α = 77.205 (4)°	*T* = 293 K
β = 84.015 (4)°	Parallelpiped, colourless
γ = 89.802 (4)°	0.4 × 0.2 × 0.15 mm
*V* = 1693.55 (14) Å^3^	

### Data collection

**Table:**

Agilent Xcalibur Eos diffractometer	5985 independent reflections
Radiation source: Enhance (Mo) X-ray Source	4876 reflections with *I* > 2σ(*I*)
Graphite monochromator	*R*_int_ = 0.041
Detector resolution: 16.0534 pixels mm^-1^	θ_max_ = 25.0°, θ_min_ = 3.1°
ω scans	*h* = −9→5
Absorption correction: multi-scan (*CrysAlis PRO*; Agilent, 2010)	*k* = −13→13
*T*_min_ = 0.122, *T*_max_ = 0.246	*l* = −22→22
11206 measured reflections	

### Refinement

**Table:**

Refinement on *F*^2^	Primary atom site location: structure-invariant direct methods
Least-squares matrix: full	Secondary atom site location: difference Fourier map
*R*[*F*^2^ > 2σ(*F*^2^)] = 0.033	Hydrogen site location: inferred from neighbouring sites
*wR*(*F*^2^) = 0.066	H-atom parameters constrained
*S* = 1.02	*w* = 1/[σ^2^(*F*_o_^2^) + (0.0191*P*)^2^] where *P* = (*F*_o_^2^ + 2*F*_c_^2^)/3
5985 reflections	(Δ/σ)_max_ < 0.001
419 parameters	Δρ_max_ = 0.65 e Å^−^^3^
0 restraints	Δρ_min_ = −1.13 e Å^−^^3^

### Special details

**Table:**

Geometry. All esds (except the esd in the dihedral angle between two l.s. planes) are estimated using the full covariance matrix. The cell esds are taken into account individually in the estimation of esds in distances, angles and torsion angles; correlations between esds in cell parameters are only used when they are defined by crystal symmetry. An approximate (isotropic) treatment of cell esds is used for estimating esds involving l.s. planes.
Refinement. Refinement of *F*^2^ against ALL reflections. The weighted *R*-factor *wR* and goodness of fit *S* are based on *F*^2^, conventional *R*-factors *R* are based on *F*, with *F* set to zero for negative *F*^2^. The threshold expression of *F*^2^ > σ(*F*^2^) is used only for calculating *R*-factors(gt) *etc*. and is not relevant to the choice of reflections for refinement. *R*-factors based on *F*^2^ are statistically about twice as large as those based on *F*, and *R*- factors based on ALL data will be even larger.

### Fractional atomic coordinates and isotropic or equivalent isotropic displacement parameters (Å^2^)

**Table:**

	*x*	*y*	*z*	*U*_iso_*/*U*_eq_	
Hg1	0.18189 (3)	0.13738 (2)	0.159189 (11)	0.04673 (8)	
Hg2	0.39752 (3)	0.29806 (2)	0.364029 (11)	0.05009 (8)	
S3	0.1361 (2)	0.30784 (17)	0.30774 (8)	0.0606 (5)	
S4	0.6423 (2)	0.18246 (17)	0.35203 (10)	0.0698 (5)	
S1	0.0329 (2)	−0.00199 (18)	0.26099 (8)	0.0716 (6)	
S2	0.3866 (2)	0.30366 (18)	0.13101 (10)	0.0722 (6)	
N5	0.4455 (5)	0.5081 (4)	0.3585 (2)	0.0404 (11)	
N6	0.2922 (6)	0.3419 (4)	0.4762 (2)	0.0447 (12)	
N2	0.0222 (5)	0.2220 (4)	0.0616 (2)	0.0322 (10)	
N1	0.2267 (5)	0.0263 (4)	0.0651 (2)	0.0310 (10)	
C11	0.1771 (6)	0.0853 (4)	−0.0005 (2)	0.0290 (11)	
C28	0.2868 (7)	0.4619 (6)	0.4772 (3)	0.0436 (14)	
C10	−0.0780 (7)	0.3145 (5)	0.0613 (3)	0.0403 (13)	
C12	0.0728 (6)	0.1882 (4)	−0.0015 (2)	0.0302 (11)	
C7	0.0207 (6)	0.2508 (5)	−0.0687 (3)	0.0370 (13)	
C27	0.3688 (7)	0.5490 (5)	0.4153 (3)	0.0411 (14)	
N4	0.3853 (7)	0.3735 (5)	−0.0213 (3)	0.0690 (16)	
C17	0.5215 (7)	0.5864 (6)	0.3020 (3)	0.0513 (16)	
C1	0.3147 (6)	−0.0736 (5)	0.0678 (3)	0.0387 (13)	
C16	0.3852 (7)	0.3451 (5)	0.0410 (4)	0.0497 (15)	
C4	0.2219 (6)	0.0459 (5)	−0.0656 (3)	0.0376 (13)	
C3	0.3154 (6)	−0.0589 (5)	−0.0603 (3)	0.0438 (14)	
H3A	0.3468	−0.0883	−0.1017	0.053*	
C23	0.2056 (8)	0.5034 (6)	0.5351 (3)	0.0526 (16)	
C2	0.3609 (6)	−0.1184 (5)	0.0055 (3)	0.0486 (15)	
H2A	0.4224	−0.1885	0.0089	0.058*	
C8	−0.0848 (7)	0.3477 (5)	−0.0666 (3)	0.0479 (15)	
H8A	−0.1211	0.3912	−0.1096	0.057*	
C6	0.0741 (7)	0.2105 (6)	−0.1336 (3)	0.0464 (15)	
H6A	0.0423	0.2528	−0.1779	0.056*	
C5	0.1695 (7)	0.1125 (6)	−0.1319 (3)	0.0461 (15)	
H5A	0.2016	0.0880	−0.1750	0.055*	
C9	−0.1353 (7)	0.3796 (5)	−0.0029 (3)	0.0490 (15)	
H9A	−0.2068	0.4437	−0.0019	0.059*	
C19	0.4423 (8)	0.7529 (6)	0.3558 (4)	0.0645 (19)	
H19A	0.4405	0.8359	0.3538	0.077*	
C15	0.1360 (8)	0.0123 (6)	0.3288 (3)	0.0556 (17)	
C14	−0.1309 (7)	0.3473 (5)	0.1335 (3)	0.0568 (17)	
H14A	−0.1896	0.2797	0.1658	0.085*	
H14B	−0.2014	0.4162	0.1258	0.085*	
H14C	−0.0353	0.3668	0.1546	0.085*	
C20	0.3639 (7)	0.6725 (6)	0.4161 (3)	0.0488 (15)	
N8	0.5652 (8)	0.0478 (6)	0.2516 (3)	0.0817 (19)	
C18	0.5223 (8)	0.7107 (6)	0.2993 (3)	0.0613 (18)	
H18A	0.5765	0.7646	0.2594	0.074*	
C31	0.1355 (8)	0.4561 (8)	0.2732 (3)	0.0620 (19)	
C13	0.3626 (7)	−0.1385 (5)	0.1401 (3)	0.0534 (16)	
H13A	0.4021	−0.0807	0.1649	0.080*	
H13B	0.4481	−0.1947	0.1331	0.080*	
H13C	0.2685	−0.1819	0.1687	0.080*	
C32	0.5939 (8)	0.1038 (6)	0.2922 (3)	0.0562 (16)	
C26	0.2191 (8)	0.2611 (6)	0.5327 (3)	0.0579 (18)	
N3	0.2032 (8)	0.0173 (6)	0.3789 (3)	0.084 (2)	
N7	0.1302 (8)	0.5602 (6)	0.2495 (3)	0.084 (2)	
C29	0.6022 (9)	0.5361 (6)	0.2401 (3)	0.075 (2)	
H29A	0.6589	0.4633	0.2591	0.112*	
H29B	0.6795	0.5950	0.2103	0.112*	
H29C	0.5195	0.5179	0.2113	0.112*	
C22	0.2020 (9)	0.6313 (7)	0.5327 (4)	0.070 (2)	
H22A	0.1451	0.6589	0.5709	0.085*	
C21	0.2791 (9)	0.7110 (7)	0.4766 (4)	0.068 (2)	
H21A	0.2775	0.7932	0.4769	0.082*	
C25	0.1346 (9)	0.2991 (7)	0.5926 (3)	0.072 (2)	
H25A	0.0827	0.2419	0.6315	0.086*	
C24	0.1290 (7)	0.4172 (5)	0.5935 (2)	0.069 (2)	
H24A	0.0740	0.4419	0.6332	0.082*	
C30	0.2305 (7)	0.1283 (5)	0.5324 (2)	0.096 (3)	
H30A	0.1531	0.1078	0.5019	0.144*	
H30B	0.2055	0.0810	0.5811	0.144*	
H30C	0.3400	0.1112	0.5140	0.144*	

### Atomic displacement parameters (Å^2^)

**Table:**

	*U*^11^	*U*^22^	*U*^33^	*U*^12^	*U*^13^	*U*^23^
Hg1	0.05436 (16)	0.04869 (16)	0.03648 (13)	0.00370 (12)	−0.00277 (11)	−0.00905 (11)
Hg2	0.05948 (17)	0.04426 (16)	0.04687 (14)	0.01347 (12)	−0.00141 (12)	−0.01299 (11)
S3	0.0617 (11)	0.0717 (13)	0.0550 (9)	0.0052 (9)	−0.0130 (9)	−0.0248 (9)
S4	0.0627 (12)	0.0703 (13)	0.0932 (12)	0.0275 (9)	−0.0289 (10)	−0.0448 (11)
S1	0.0765 (13)	0.0860 (14)	0.0467 (9)	−0.0330 (11)	−0.0061 (9)	−0.0024 (9)
S2	0.0691 (12)	0.0768 (14)	0.0767 (12)	−0.0223 (10)	−0.0121 (10)	−0.0277 (10)
N5	0.046 (3)	0.040 (3)	0.036 (2)	0.006 (2)	−0.005 (2)	−0.009 (2)
N6	0.055 (3)	0.051 (3)	0.029 (2)	0.001 (3)	−0.006 (2)	−0.009 (2)
N2	0.026 (2)	0.032 (3)	0.040 (2)	0.0019 (19)	0.0036 (19)	−0.014 (2)
N1	0.024 (2)	0.025 (2)	0.042 (2)	0.0009 (19)	−0.001 (2)	−0.005 (2)
C11	0.022 (3)	0.028 (3)	0.037 (3)	−0.004 (2)	0.006 (2)	−0.010 (2)
C28	0.040 (3)	0.056 (4)	0.040 (3)	0.009 (3)	−0.017 (3)	−0.017 (3)
C10	0.034 (3)	0.039 (3)	0.054 (3)	0.002 (3)	−0.003 (3)	−0.023 (3)
C12	0.024 (3)	0.031 (3)	0.037 (3)	−0.002 (2)	0.000 (2)	−0.012 (2)
C7	0.029 (3)	0.038 (3)	0.041 (3)	−0.004 (2)	−0.003 (3)	−0.005 (3)
C27	0.045 (4)	0.040 (4)	0.041 (3)	0.008 (3)	−0.012 (3)	−0.010 (3)
N4	0.059 (4)	0.055 (4)	0.085 (4)	0.006 (3)	0.010 (4)	−0.006 (3)
C17	0.050 (4)	0.052 (4)	0.050 (3)	−0.002 (3)	−0.002 (3)	−0.007 (3)
C1	0.020 (3)	0.034 (3)	0.057 (3)	−0.003 (2)	0.003 (3)	−0.003 (3)
C16	0.031 (3)	0.039 (4)	0.081 (4)	0.008 (3)	0.005 (3)	−0.022 (4)
C4	0.026 (3)	0.045 (4)	0.045 (3)	−0.010 (3)	0.008 (2)	−0.022 (3)
C3	0.032 (3)	0.044 (4)	0.061 (4)	−0.007 (3)	0.010 (3)	−0.030 (3)
C23	0.052 (4)	0.072 (5)	0.042 (3)	0.010 (3)	−0.010 (3)	−0.028 (3)
C2	0.031 (3)	0.041 (4)	0.078 (4)	0.008 (3)	0.009 (3)	−0.030 (3)
C8	0.042 (4)	0.045 (4)	0.054 (3)	0.002 (3)	−0.014 (3)	0.000 (3)
C6	0.041 (4)	0.067 (5)	0.029 (3)	−0.010 (3)	−0.003 (3)	−0.005 (3)
C5	0.037 (3)	0.067 (5)	0.038 (3)	−0.013 (3)	0.007 (3)	−0.024 (3)
C9	0.041 (4)	0.039 (4)	0.070 (4)	0.009 (3)	−0.013 (3)	−0.015 (3)
C19	0.080 (5)	0.040 (4)	0.077 (5)	0.000 (4)	−0.028 (4)	−0.012 (4)
C15	0.065 (4)	0.049 (4)	0.047 (3)	0.000 (3)	0.012 (3)	−0.006 (3)
C14	0.057 (4)	0.052 (4)	0.069 (4)	0.020 (3)	−0.005 (3)	−0.029 (3)
C20	0.049 (4)	0.047 (4)	0.057 (4)	0.008 (3)	−0.020 (3)	−0.018 (3)
N8	0.095 (5)	0.076 (5)	0.081 (4)	0.003 (4)	0.010 (4)	−0.041 (4)
C18	0.074 (5)	0.047 (4)	0.057 (4)	−0.008 (4)	−0.013 (4)	0.004 (3)
C31	0.057 (4)	0.098 (6)	0.036 (3)	0.025 (4)	−0.016 (3)	−0.021 (4)
C13	0.041 (4)	0.044 (4)	0.073 (4)	0.009 (3)	−0.008 (3)	−0.007 (3)
C32	0.060 (4)	0.044 (4)	0.060 (4)	0.012 (3)	0.012 (3)	−0.011 (3)
C26	0.064 (4)	0.068 (5)	0.038 (3)	−0.006 (4)	−0.006 (3)	−0.005 (3)
N3	0.108 (5)	0.088 (5)	0.057 (3)	−0.006 (4)	−0.021 (4)	−0.013 (3)
N7	0.113 (6)	0.076 (5)	0.062 (4)	0.042 (4)	−0.023 (4)	−0.009 (4)
C29	0.090 (6)	0.070 (5)	0.055 (4)	−0.005 (4)	0.021 (4)	−0.007 (4)
C22	0.073 (5)	0.088 (6)	0.069 (4)	0.022 (4)	−0.016 (4)	−0.051 (4)
C21	0.079 (5)	0.062 (5)	0.079 (5)	0.016 (4)	−0.026 (4)	−0.043 (4)
C25	0.083 (5)	0.094 (6)	0.033 (3)	−0.009 (5)	0.004 (3)	−0.007 (4)
C24	0.070 (5)	0.099 (6)	0.042 (4)	0.004 (4)	0.002 (3)	−0.029 (4)
C30	0.154 (9)	0.067 (6)	0.053 (4)	−0.017 (6)	0.011 (5)	0.004 (4)

### Geometric parameters (Å, º)

**Table:**

Hg1—N1	2.396 (4)	C3—H3A	0.9300
Hg1—N2	2.395 (4)	C23—C24	1.395 (7)
Hg1—S1	2.4201 (16)	C23—C22	1.436 (9)
Hg1—S2	2.4488 (16)	C2—H2A	0.9300
Hg2—N5	2.384 (4)	C8—C9	1.359 (7)
Hg2—N6	2.362 (4)	C8—H8A	0.9300
Hg2—S3	2.4741 (16)	C6—C5	1.348 (8)
Hg2—S4	2.4013 (18)	C6—H6A	0.9300
S3—C31	1.658 (8)	C5—H5A	0.9300
S4—C32	1.666 (7)	C9—H9A	0.9300
S1—C15	1.644 (7)	C19—C18	1.371 (9)
S2—C16	1.666 (7)	C19—C20	1.390 (8)
N5—C17	1.327 (7)	C19—H19A	0.9300
N5—C27	1.357 (6)	C15—N3	1.156 (7)
N6—C26	1.332 (7)	C14—H14A	0.9600
N6—C28	1.361 (7)	C14—H14B	0.9600
N2—C10	1.324 (7)	C14—H14C	0.9600
N2—C12	1.360 (6)	C20—C21	1.426 (8)
N1—C1	1.330 (7)	N8—C32	1.140 (7)
N1—C11	1.374 (6)	C18—H18A	0.9300
C11—C4	1.413 (6)	C31—N7	1.164 (8)
C11—C12	1.436 (7)	C13—H13A	0.9600
C28—C23	1.392 (8)	C13—H13B	0.9600
C28—C27	1.455 (7)	C13—H13C	0.9600
C10—C9	1.400 (7)	C26—C25	1.415 (9)
C10—C14	1.514 (7)	C26—C30	1.504 (8)
C12—C7	1.418 (6)	C29—H29A	0.9600
C7—C8	1.395 (8)	C29—H29B	0.9600
C7—C6	1.430 (7)	C29—H29C	0.9600
C27—C20	1.400 (7)	C22—C21	1.333 (9)
N4—C16	1.154 (7)	C22—H22A	0.9300
C17—C18	1.394 (8)	C21—H21A	0.9300
C17—C29	1.504 (8)	C25—C24	1.339 (8)
C1—C2	1.401 (7)	C25—H25A	0.9300
C1—C13	1.493 (7)	C24—H24A	0.9300
C4—C3	1.397 (8)	C30—H30A	0.9600
C4—C5	1.421 (7)	C30—H30B	0.9600
C3—C2	1.366 (7)	C30—H30C	0.9600
			
N2—Hg1—N1	70.31 (13)	C1—C2—H2A	120.0
N2—Hg1—S1	115.08 (10)	C9—C8—C7	121.1 (5)
N1—Hg1—S1	105.19 (10)	C9—C8—H8A	119.4
N2—Hg1—S2	95.17 (10)	C7—C8—H8A	119.4
N1—Hg1—S2	107.09 (10)	C5—C6—C7	121.3 (5)
S1—Hg1—S2	141.59 (6)	C5—C6—H6A	119.4
N6—Hg2—N5	71.23 (15)	C7—C6—H6A	119.4
N6—Hg2—S4	122.12 (12)	C6—C5—C4	121.2 (5)
N5—Hg2—S4	114.77 (12)	C6—C5—H5A	119.4
N6—Hg2—S3	98.11 (12)	C4—C5—H5A	119.4
N5—Hg2—S3	100.38 (11)	C8—C9—C10	118.9 (6)
S4—Hg2—S3	132.60 (6)	C8—C9—H9A	120.6
C31—S3—Hg2	98.6 (2)	C10—C9—H9A	120.6
C32—S4—Hg2	101.1 (2)	C18—C19—C20	120.5 (6)
C15—S1—Hg1	101.9 (2)	C18—C19—H19A	119.8
C16—S2—Hg1	100.0 (2)	C20—C19—H19A	119.8
C17—N5—C27	119.7 (5)	N3—C15—S1	176.3 (6)
C17—N5—Hg2	125.3 (4)	C10—C14—H14A	109.5
C27—N5—Hg2	114.4 (3)	C10—C14—H14B	109.5
C26—N6—C28	119.2 (5)	H14A—C14—H14B	109.5
C26—N6—Hg2	124.8 (4)	C10—C14—H14C	109.5
C28—N6—Hg2	115.3 (4)	H14A—C14—H14C	109.5
C10—N2—C12	120.1 (4)	H14B—C14—H14C	109.5
C10—N2—Hg1	124.5 (3)	C19—C20—C27	117.0 (6)
C12—N2—Hg1	113.5 (3)	C19—C20—C21	123.0 (6)
C1—N1—C11	118.9 (4)	C27—C20—C21	120.0 (6)
C1—N1—Hg1	126.2 (3)	C19—C18—C17	119.4 (6)
C11—N1—Hg1	114.1 (3)	C19—C18—H18A	120.3
N1—C11—C4	122.1 (5)	C17—C18—H18A	120.3
N1—C11—C12	118.1 (4)	N7—C31—S3	178.0 (7)
C4—C11—C12	119.8 (5)	C1—C13—H13A	109.5
N6—C28—C23	122.1 (6)	C1—C13—H13B	109.5
N6—C28—C27	118.4 (5)	H13A—C13—H13B	109.5
C23—C28—C27	119.4 (6)	C1—C13—H13C	109.5
N2—C10—C9	121.8 (5)	H13A—C13—H13C	109.5
N2—C10—C14	117.6 (5)	H13B—C13—H13C	109.5
C9—C10—C14	120.6 (5)	N8—C32—S4	177.9 (7)
N2—C12—C7	121.1 (5)	N6—C26—C25	120.7 (6)
N2—C12—C11	119.8 (4)	N6—C26—C30	118.9 (5)
C7—C12—C11	119.0 (4)	C25—C26—C30	120.4 (6)
C8—C7—C12	117.0 (5)	C17—C29—H29A	109.5
C8—C7—C6	123.7 (5)	C17—C29—H29B	109.5
C12—C7—C6	119.3 (5)	H29A—C29—H29B	109.5
N5—C27—C20	122.2 (5)	C17—C29—H29C	109.5
N5—C27—C28	119.1 (5)	H29A—C29—H29C	109.5
C20—C27—C28	118.7 (5)	H29B—C29—H29C	109.5
N5—C17—C18	121.1 (6)	C21—C22—C23	120.9 (6)
N5—C17—C29	117.4 (6)	C21—C22—H22A	119.5
C18—C17—C29	121.5 (6)	C23—C22—H22A	119.5
N1—C1—C2	121.7 (5)	C22—C21—C20	121.2 (6)
N1—C1—C13	118.0 (5)	C22—C21—H21A	119.4
C2—C1—C13	120.4 (5)	C20—C21—H21A	119.4
N4—C16—S2	179.5 (6)	C24—C25—C26	120.1 (6)
C3—C4—C11	117.1 (5)	C24—C25—H25A	119.9
C3—C4—C5	123.5 (5)	C26—C25—H25A	119.9
C11—C4—C5	119.4 (5)	C25—C24—C23	120.1 (5)
C2—C3—C4	120.2 (5)	C25—C24—H24A	119.9
C2—C3—H3A	119.9	C23—C24—H24A	119.9
C4—C3—H3A	119.9	C26—C30—H30A	109.5
C28—C23—C24	117.7 (6)	C26—C30—H30B	109.5
C28—C23—C22	119.7 (6)	H30A—C30—H30B	109.5
C24—C23—C22	122.5 (6)	C26—C30—H30C	109.5
C3—C2—C1	120.0 (5)	H30A—C30—H30C	109.5
C3—C2—H2A	120.0	H30B—C30—H30C	109.5
			
N6—Hg2—S3—C31	85.6 (3)	Hg2—N5—C27—C28	8.4 (6)
N5—Hg2—S3—C31	13.3 (2)	N6—C28—C27—N5	0.9 (7)
S4—Hg2—S3—C31	−125.1 (2)	C23—C28—C27—N5	−179.0 (5)
N6—Hg2—S4—C32	143.6 (3)	N6—C28—C27—C20	−179.9 (5)
N5—Hg2—S4—C32	−133.8 (2)	C23—C28—C27—C20	0.2 (8)
S3—Hg2—S4—C32	0.2 (3)	C27—N5—C17—C18	−0.9 (8)
N2—Hg1—S1—C15	−152.7 (3)	Hg2—N5—C17—C18	170.2 (4)
N1—Hg1—S1—C15	132.3 (3)	C27—N5—C17—C29	−179.0 (5)
S2—Hg1—S1—C15	−14.0 (3)	Hg2—N5—C17—C29	−8.0 (7)
N2—Hg1—S2—C16	−31.0 (2)	C11—N1—C1—C2	1.5 (7)
N1—Hg1—S2—C16	40.0 (2)	Hg1—N1—C1—C2	−167.2 (3)
S1—Hg1—S2—C16	−174.1 (2)	C11—N1—C1—C13	−178.0 (4)
N6—Hg2—N5—C17	179.0 (5)	Hg1—N1—C1—C13	13.4 (6)
S4—Hg2—N5—C17	61.5 (5)	Hg1—S2—C16—N4	−115 (83)
S3—Hg2—N5—C17	−85.9 (4)	N1—C11—C4—C3	2.0 (7)
N6—Hg2—N5—C27	−9.6 (3)	C12—C11—C4—C3	−176.3 (4)
S4—Hg2—N5—C27	−127.1 (3)	N1—C11—C4—C5	−178.6 (4)
S3—Hg2—N5—C27	85.5 (4)	C12—C11—C4—C5	3.1 (7)
N5—Hg2—N6—C26	−179.7 (5)	C11—C4—C3—C2	−0.5 (7)
S4—Hg2—N6—C26	−71.7 (5)	C5—C4—C3—C2	−179.8 (5)
S3—Hg2—N6—C26	82.0 (5)	N6—C28—C23—C24	0.3 (8)
N5—Hg2—N6—C28	10.1 (3)	C27—C28—C23—C24	−179.8 (5)
S4—Hg2—N6—C28	118.1 (4)	N6—C28—C23—C22	−179.1 (5)
S3—Hg2—N6—C28	−88.1 (4)	C27—C28—C23—C22	0.8 (8)
N1—Hg1—N2—C10	178.6 (4)	C4—C3—C2—C1	−0.5 (8)
S1—Hg1—N2—C10	80.6 (4)	N1—C1—C2—C3	0.0 (8)
S2—Hg1—N2—C10	−75.1 (4)	C13—C1—C2—C3	179.5 (5)
N1—Hg1—N2—C12	−17.2 (3)	C12—C7—C8—C9	−0.3 (7)
S1—Hg1—N2—C12	−115.2 (3)	C6—C7—C8—C9	178.0 (5)
S2—Hg1—N2—C12	89.1 (3)	C8—C7—C6—C5	−176.6 (5)
N2—Hg1—N1—C1	−174.6 (4)	C12—C7—C6—C5	1.7 (8)
S1—Hg1—N1—C1	−62.9 (4)	C7—C6—C5—C4	−0.5 (8)
S2—Hg1—N1—C1	96.0 (4)	C3—C4—C5—C6	177.5 (5)
N2—Hg1—N1—C11	16.3 (3)	C11—C4—C5—C6	−1.9 (8)
S1—Hg1—N1—C11	128.0 (3)	C7—C8—C9—C10	0.9 (8)
S2—Hg1—N1—C11	−73.1 (3)	N2—C10—C9—C8	−0.9 (8)
C1—N1—C11—C4	−2.5 (6)	C14—C10—C9—C8	179.8 (5)
Hg1—N1—C11—C4	167.4 (3)	Hg1—S1—C15—N3	−175 (11)
C1—N1—C11—C12	175.8 (4)	C18—C19—C20—C27	−1.0 (9)
Hg1—N1—C11—C12	−14.2 (5)	C18—C19—C20—C21	179.9 (6)
C26—N6—C28—C23	−0.7 (8)	N5—C27—C20—C19	−0.3 (8)
Hg2—N6—C28—C23	170.0 (4)	C28—C27—C20—C19	−179.4 (5)
C26—N6—C28—C27	179.4 (5)	N5—C27—C20—C21	178.9 (5)
Hg2—N6—C28—C27	−9.9 (6)	C28—C27—C20—C21	−0.3 (8)
C12—N2—C10—C9	0.3 (7)	C20—C19—C18—C17	1.3 (10)
Hg1—N2—C10—C9	163.5 (4)	N5—C17—C18—C19	−0.4 (9)
C12—N2—C10—C14	179.6 (4)	C29—C17—C18—C19	177.7 (6)
Hg1—N2—C10—C14	−17.2 (6)	Hg2—S3—C31—N7	−141 (17)
C10—N2—C12—C7	0.3 (7)	Hg2—S4—C32—N8	−172 (17)
Hg1—N2—C12—C7	−164.7 (3)	C28—N6—C26—C25	0.9 (9)
C10—N2—C12—C11	−177.9 (4)	Hg2—N6—C26—C25	−168.9 (5)
Hg1—N2—C12—C11	17.1 (5)	C28—N6—C26—C30	−177.9 (5)
N1—C11—C12—N2	−2.1 (6)	Hg2—N6—C26—C30	12.3 (7)
C4—C11—C12—N2	176.3 (4)	C28—C23—C22—C21	−1.8 (10)
N1—C11—C12—C7	179.7 (4)	C24—C23—C22—C21	178.8 (6)
C4—C11—C12—C7	−1.9 (7)	C23—C22—C21—C20	1.7 (10)
N2—C12—C7—C8	−0.3 (7)	C19—C20—C21—C22	178.4 (6)
C11—C12—C7—C8	178.0 (4)	C27—C20—C21—C22	−0.7 (10)
N2—C12—C7—C6	−178.7 (4)	N6—C26—C25—C24	−0.8 (10)
C11—C12—C7—C6	−0.4 (7)	C30—C26—C25—C24	178.0 (7)
C17—N5—C27—C20	1.2 (8)	C26—C25—C24—C23	0.4 (10)
Hg2—N5—C27—C20	−170.8 (4)	C28—C23—C24—C25	−0.2 (9)
C17—N5—C27—C28	−179.6 (5)	C22—C23—C24—C25	179.3 (6)
